# Anomaly-based intrusion detection on benchmark datasets for network security: a comprehensive evaluation

**DOI:** 10.1038/s41598-026-38317-w

**Published:** 2026-03-09

**Authors:** L. K. Suresh Kumar, Srihith Reddy Nethi, Ravi Uyyala, Padmavathi Vurubindi, Sujatha Canavoy Narahari, Ashok Kumar Das, Vivekananda Bhat K, Mohammed J. F. Alenazi

**Affiliations:** 1 Department of Computer Science and Engineering, University College of Engineering, Osmania University, Hyderabad, 500 007 India; 2 Department of Computer Science and Engineering, Chaitanya Bharathi Institute of Technology, Hyderabad, 500 075 India; 3 Department of ECE, Sreenidhi Institute of Science and Technology, Hyderabad, 501 302 India; 4 Center for Security, Theory and Algorithmic Research, International Institute of Information Technology, Hyderabad, 500 032 India; 5 Department of Computer Science and Engineering, College of Informatics, Korea University, Seongbuk-gu, Seoul 02841 South Korea; 6 Manipal Institute of Technology, Manipal Academy of Higher Education, Manipal, Karnataka 576104 India; 7 Department of Computer Engineering, College of Computer and Information Sciences (CCIS), King Saud University, 11451 Riyadh, Saudi Arabia

**Keywords:** Intrusion Detection System (IDS), Multi-class classification, Network security, Cybersecurity, Network attacks, Computational biology and bioinformatics, Engineering, Mathematics and computing

## Abstract

This study discusses two widely-recognized deep learning approaches for network intrusion detection: a Deep Neural Network (DNN) and a Recurrent Neural Network (RNN). Both models are trained and evaluated on three widely used benchmark datasets: KDDCup99, NSL-KDD (each with five classes), and UNSW-NB15 (ten classes). Multiple optimizers, including Adam, SGD, Adamax, AdamW, and Adadelta, are then explored, with Adam consistently providing the best performance. CrossEntropyLoss is found to be the most effective loss function for these multi-class classification tasks. Designed to automatically learn and extract relevant features from raw data, the models reduce reliance on manual feature engineering. Performance is assessed using accuracy, precision, recall, F1-score, and false positive rate. Experimental results show that both models achieve over 99% accuracy on KDDCup99, with improved detection rates and false positive rates below 1% for KDDCup99 and NSL-KDD. On the more complex UNSW-NB15 dataset, false positive rates also remain under 8%, demonstrating the models’ robustness and generalizability across diverse intrusion scenarios.

## Introduction

The growing reliance on the internet has increased exposure to cyber threats, making intrusion detection a critical security requirement. An “Intrusion Detection System (IDS)” monitors network traffic and identifies suspicious or abnormal activity. IDSs can be passive, issuing alerts, or active, attempting real-time mitigation, and may suffer from false positives. Traditional IDS tools, such as Snort and Zeek, which rely on signatures and rules, struggle to handle today’s complex and evolving attacks, especially zero-day exploits. To address these limitations, modern IDS frameworks increasingly adopt deep learning, which enables systems to automatically learn patterns of normal and malicious behaviors. This shift provides more adaptive, accurate, and scalable detection capabilities for real-world cybersecurity environments.

### IDS methodologies

An IDS can be defined on the basis of techniques or development strategies.

#### Technique-based approaches

Here, we discuss the following approaches.

*Misuse-based detection:* This method, which is also known as misuse detection, is predicated on a previously established database of acknowledged attack signatures or patterns. Each signature is a unique fingerprint of a specific type of malicious behavior that has been previously identified and stored in the system. When new network traffic is analyzed, it is compared against this database to detect any matches. If a match is found, the system flags the event as a known attack and may take appropriate action, such as blacklisting the source or destination node^1^. Signature-based IDS offers high accuracy and low false positives when dealing with known threats, making it computationally efficient and reliable in environments with predictable attack patterns. However, their primary limitation lies in their inability to detect novel or zero-day attacks, since no predefined signature exists for such exploits. This leaves them vulnerable to sophisticated and evolving threats that target previously unknown system weaknesses.

*Anomaly dependent detection:* The IDS, which works on anomalies, works on a fundamentally different principle. Rather than relying on a database of known threats, it establishes a baseline of what constitutes “normal” behavior within the network–typically derived through statistical analysis, machine learning, or historical observation^2^. Any deviation from this learned normal behavior is flagged as potentially malicious^3^. Anomaly-based IDS is particularly effective at detecting zero-day or previously unknown attacks, since they do not depend on predefined threat signatures. By modeling normal behavior, they can identify subtle deviations that may signal intrusions, malware, or insider threats. However, these systems often suffer from higher false positive rates, especially during the learning phase or in dynamic environments where “normal” behavior shifts frequently. They also demand greater computational resources and careful tuning to reduce misclassifications.

*Hybrid-based detection:* Hybrid IDS combines the accuracy of signature-based detection with the flexibility of anomaly-based methods. Typically, traffic is first checked against known signatures; if no match is found, anomaly analysis is applied. This approach reduces false alarms while still detecting zero-day attacks, offering broader coverage than either method alone. With advances in deep learning and ensemble techniques, hybrid IDSs have gained importance for their ability to fuse features effectively and withstand diverse attack scenarios. Their balanced design makes them well-suited for deployment in complex, real-time network environments.

#### IDS based on development strategy

IDS can be categorized based on the underlying development strategy, i.e., how the system is built to process and detect intrusions. Broadly, these systems can be classified into two major types: 1) rule-based systems and 2) learning-based systems. This classification is centered around whether the system depends on predefined rules or evolves through data-driven learning methods.

*Rule-based systems:* Rule-based IDSs are among the most traditional and widely deployed intrusion detection approaches. Examples include well-established open-source platforms such as *Snort* and *Zeek (formerly Bro)*. These systems operate based on a predefined set of human-written rules or signatures that represent known patterns of malicious activity. These rules are typically constructed using domain knowledge, previous incident logs, or known attack signatures stored in structured rule files or configuration scripts^4^. Rule-based IDS detects threats by matching traffic against predefined attack signatures, issuing alerts or responses when a match occurs. They are effective for known attacks but struggle with zero-day exploits and evolving threats. Constant rule updates also demand heavy manual effort, making them less practical in today’s fast-changing, high-traffic networks.

*Machine learning/deep learning-based systems:* Learning-based IDS uses machine learning and deep learning to detect intrusions by modeling traffic patterns and system behavior, enabling recognition of both known and evolving threats. Classical ML methods (e.g., Random Forests, SVM (Support Vector Machine), KNN (K-Nearest Neighbors)) achieve strong accuracy on datasets, like NSL-KDD^5^ and UNSW-NB15^6^, while deep learning models, such as RNN (Recurrent Neural Network), LSTM (Long Short-Term Memory), Convolutional Neural Network (CNN), and DNN (Deep Neural Network), further enhance detection by capturing temporal or hierarchical features. These systems are adaptive but face challenges, including reliance on large labeled datasets, high training costs, and potential overfitting. To balance precision and adaptability, modern IDS often adopts hybrid designs that integrate rule-based and learning-based approaches for more resilient protection.

### AI/ML and DL

Artificial Intelligence (AI) enables machines to replicate human-like reasoning, decision-making, and problem-solving, with Artificial Neural Networks (ANNs) serving as the primary inspiration. Within AI, “Machine Learning (ML)” allows systems to learn patterns from data instead of relying on fixed rules. Algorithms, such as Support Vector Machines (SVM), Decision Trees, and “K-Nearest Neighbors (KNN)” have been widely applied across domains, offering adaptability and generalization to unseen data. Deep Learning (DL), a specialized branch of ML, uses multi-layered neural networks to automatically extract hierarchical features from complex data such as images, text, and network traffic. Advances in architectures like CNNs, RNNs, LSTMs, and Transformers, coupled with big data and GPU acceleration, have made DL particularly powerful for high-dimensional problems. In IDS, AI-driven methods represent a shift from static, rule-based detection to adaptive learning. Models trained on benchmark datasets (e.g., KDDCup99^7^, NSL-KDD, UNSW-NB15) can identify anomalies, novel threats, and multi-stage attacks, making AI, ML, and DL critical foundations for building scalable and intelligent IDS.

### Artificial neural network (ANN)

ANNs have become a defining centerpiece of AI apps, especially in fields like Natural Language Processing (NLP), image recognition, and cybersecurity. Among the various architectures, DNNs and RNNs are among the most widely adopted models because of their ability to learn complex patterns and represent a range of representations.

#### Deep neural network (DNN)

It is an ANN with two or more hidden layers between the input and output. These layers consist of neurons that apply weighted sums and non-linear activation functions, enabling the network to transform inputs through multiple stages. This depth allows the model to learn hierarchical features–lower layers capture simple patterns, while deeper layers extract more abstract representations. Non-linear activations such as ReLU are central to DNNs, as they allow the network to capture complex, non-linear relationships and generalize better to unseen data. This makes DNNs powerful in scenarios where linear models fail to detect subtle patterns. DNNs are highly flexible, as their architecture can be tuned through choices of depth, neurons, activation functions, and optimizers. However, deeper models demand more computational resources and are susceptible to overfitting, particularly on small or noisy datasets. Regularization techniques like dropout, batch normalization, and early stopping are often applied to address these challenges. For intrusion detection, DNNs are especially valuable because they can automatically extract relevant features and capture complex interactions among attributes. This makes them well-suited for modern IDS datasets such as NSL-KDD and UNSW-NB15, which are multi-dimensional and multi-class in nature.

#### Recurrent neural networks (RNN)

RNNs are a type of neural network made for processing and computing sequential data. Unlike traditional feedforward networks such as DNNs, which assume that inputs are different from one another, RNNs introduce the concept of “memory” by maintaining a hidden state that is updated at each time. This hidden state carries forward information about previous inputs, enabling the network to identify contextual relationships and temporal dependencies within the data. Mathematically, an RNN computes the output at time step $t$ as a derivative of the input $x_t$ and the hidden state from the previous step $h_{t-1}$. This recurrent connection establishes a loop that enables information to endure across time steps:

$$h_t = \sigma (W_{hh} h_{t-1} + W_{xh} x_t + b_h), \quad y_t = W_{hy} h_t + b_y$$

where $W_{hh}, W_{xh}, W_{hy}$ are weight-matrices, $b_h, b_y$ are the bias vectors, and $\sigma$ is a non-linear activation function. RNNs are well-suited for tasks where data order matters, such as speech, language processing, and intrusion detection. In IDS, sequential traffic can reveal hidden attack patterns–benign packets in isolation may collectively indicate malicious activity. RNNs capture these temporal dependencies through their recurrent structure. Standard RNNs, however, face challenges like vanishing and exploding gradients on long sequences. Variants such as LSTMs and GRUs address this with gating mechanisms that selectively retain or discard information, enabling better modeling of long-term dependencies. In practice, DNNs and RNNs complement each other: DNNs learn spatial or feature-based hierarchies, while RNNs capture temporal behavior. For intrusion detection, DNNs excel at analyzing individual records, whereas RNNs track evolving traffic flows. Model selection depends on dataset characteristics and detection objectives.

There is a critical need for anomaly-based intrusion detection systems (IDS) that maintain a low false positive rate. Existing models often suffer from trade-offs, showing limitations either in accuracy or false positives or both. While signature-based systems are known for their low false positive rates, they lack adaptability to novel threats. The objective, therefore, is to design an anomaly-based IDS that achieves comparable reliability by leveraging deep learning techniques, combining adaptability with precision.

### Literature review

A wide range of IDS have been proposed using both ML and DL methodologies. Like previously stated, DL-based systems offer improved adaptability and higher accuracy, especially in complex network environments. This section reviews recent work on IDS models based on both ML and DL approaches to identify their strengths and limitations.

The work proposed in^8^ has built an anomaly-based IDS using Naïve Bayes classifiers and evaluated its performance on datasets such as NSL-KDD, UNSW-NB15, and CICIDS2017. Their supervised classification model got an overall 97% accuracy, but its performance declined in multi-class classification scenarios. The scheme in^9^ applied four different machine learning algorithms to the UNSW-NB15 dataset. The Support Vector Machine (SVM) classifier yielded an accuracy of 92.28%, while Naïve Bayes reached only 74.19%. Decision tree and random forest classifiers performed better, achieving 95.82% and 97.49% accuracy, respectively. The authors in^10^ used a hybrid anomaly-based IDS by using a combination of K-Means clustering, Gaussian mixture models, and ensemble supervised classifiers, including KNN and SVM. Their system achieved 99.85% accuracy with the NSL-KDD dataset and 98.27% with the KDDCup99 dataset.

Beyond traditional ML methods, deep learning techniques have also been explored. An approach^11^ employed a sparse autoencoder combined with a logistic classifier to build a DL-based IDS trained on the NSL-KDD dataset. Their system achieved an accuracy of 87.2%, demonstrating the potential of representation learning for intrusion detection. The authors in^12^ suggested one with an ANN-based IDS for the KDD-99 dataset. Their model incorporated feature reduction to boost learning efficiency and achieved category-wise accuracies of 99.93% (DoS), 98.7% (Probe), 92.54% (R2L), and 96.51% (U2R), indicating strong performance across different attack types. The authors in^13^ conducted a comprehensive evaluation of a deep feed-forward neural network with 20 hidden layers, which was employed to analyze the KDDCup99, NSL-KDD, and UNSW-NB15 datasets. Their model consistently achieved above 90% accuracy on all datasets, showcasing the suitability of deep architectures for multi-class IDS tasks, particularly in IoT environments. The authors in^14^ also evaluated DNN and RNN models on KDDCup99 and NSL-KDD, achieving 98.95% (DNN) and 98.68% (RNN) accuracy on NSL-KDD, and 98.20% (DNN) and 98.73% (RNN) on KDDCup99 using one-hot encoding, MinMaxScaler, and shallow networks.

The survey by Meliboev et al.^15^ made a set of 5 models on NSL-KDD, KDDcup99, and UNSW-NB15 datasets, each of which led to roughly 77%, 91%, and 75% accuracy by using CNN, LSTM, GRU, RNN, and a combination of CNN and LSTM. Bamber et al.^16^ compared ANN, LSTM, BiLSTM, CNN–LSTM, GRU, and BiGRU, with the CNN–LSTM hybrid achieving the highest accuracy at nearly 95%. This performance gain was credited to CNN’s spatial feature extraction and LSTM’s ability to capture temporal dependencies in network traffic.

Akter et al.^17^ introduced “SCGNet: Stacked Convolution with Gated Recurrent Unit Network,” a novel hybrid model designed for intrusion detection and attack-type classification. Evaluated on the NSL-KDD dataset, SCGNet achieved an outstanding 99.76% accuracy for attack detection, and 98.92% accuracy for multi-class attack-type classification. Azarudeen et al.^18^ proposed an intrusion detection system based on RNNs, where the dataset was partitioned into training, testing, and validation sets in a 15:3:2 ratio. The model attained a mean training accuracy of 96.52% and a validation accuracy of 97.10%. Radhi and Mohammed^19^ introduced NIDS-DL for SDN using 12 of 41 NSL-KDD features, benchmarking CNN, DNN, RNN, LSTM, and GRU for binary attack detection. The models achieved accuracies of 98.63% (CNN), 98.53% (DNN), 98.13% (RNN), 98.04% (LSTM), and 97.78% (GRU), highlighting CNN’s edge with compact inputs and suggesting hybrid CNN-RNN approaches as promising for SDN-aware NIDS.

Yin et al.^20^ compared RNN, LSTM, and GRU on NSL-KDD with feature selection, reporting accuracies of 89.6% (RNN), 92.0% (LSTM), and 91.8% (GRU). The results showed LSTM slightly outperformed GRU, and both gated models significantly surpassed vanilla RNN, underscoring their effectiveness for IDS on sequential data. Adefemi Alimi et al.^21^ proposed a refined LSTM-based IDS for “Denial-of-Service (DoS) attack detection” on NSL-KDD, achieving 96.5% training accuracy and 97.10% validation accuracy. The results show improved generalization and lower error rates compared to standard LSTM setups for DoS traffic detection.

Gharibian and Ghorbani^22^ compared the use of supervised probabilistic and predictive machine learning methods for detecting intrusions. They used two probabilistic techniques, namely Naive Bayes and Gaussian, and two predictive techniques, namely decision tree and random forests. The training data comes from different sets created from the KDD99 dataset. Their results compare how well each technique detects four types of attacks: DoS, Probe, R2L, and U2R. They also included statistical results showing how sensitive each technique is to the number of attacks in the dataset.

Tang et al.^23^ proposed a “Deep Stacking Network (DSN) for NSL-KDD intrusion detection,” by evaluating standalone classifiers–Decision Tree, KNN, DNN, and Random Forest–with the best standalone accuracy of 86.1% (Decision Tree). Stacking these classifiers in the DSN ensemble improved accuracy to 86.8%, demonstrating the benefit of ensemble fusion over standard ML approaches. Devarakonda et al.^24^ studied and compared DT, MLP, RF, and SAE on KDD-99 and NSL-KDD, with Random Forest achieving the highest accuracies of 99.41% (KDD-99) and 83.33% (NSL-KDD). DT scored 98.49%/82.02%, MLP 99.36%/81.20%, and SAE 91.13%/78.21%, showing RF’s superior performance across both datasets. Kasongo^25^ also proposed an “RNN-based IDS comparing Simple RNN, LSTM, and GRU on NSL-KDD and UNSW-NB15 using XGBoost-selected features (22 for NSL-KDD, 17 for UNSW-NB15).” GRU achieved the highest accuracies–89.3% (NSL-KDD) and 87.6% (UNSW-NB15)–surpassing LSTM (87.8%/86.2%) and RNN (86.1%/84.5%), demonstrating superior detection accuracy and generalization.

Kumar et al.^26^ applied K-means clustering to NSL-KDD, achieving 82.19% accuracy, 79.65% detection rate, and 5.4% false alarm rate. While less effective than supervised deep learning, it highlighted unsupervised learning’s value in revealing hidden structures and detecting novel attacks. An empirical study in^27^ employed CNNs for network anomaly detection on NSL-KDD, achieving 93.65% accuracy, 92.8% precision, and 91.4% recall. The results outperformed baseline ML methods like SVM and Random Forest, which demonstrate CNNs’ superior feature extraction for IDS.

Cheng et al.^28^ proposed DESC-IDS, a hybrid deep learning and evolving stream clustering system for automotive IDS, achieving 94.27% accuracy, 92.4% detection rate, and 3.1% false positive rate on benchmark vehicular datasets. The results highlight its real-time adaptability and robustness in connected vehicle networks. Farahnakian and Heikkonen^29^ proposed a deep autoencoder-based IDS on NSL-KDD, achieving 87.5% accuracy, 85.6% detection rate, and 4.9% false alarm rate. The autoencoder reduced data dimensionality while preserving attack features, demonstrating the effectiveness of unsupervised deep learning for intrusion detection.

Altunay and Albayrak^30^ proposed a hybrid CNN-LSTM IDS for UNSW-NB15, capturing spatial feature correlations with CNNs and temporal dependencies with LSTMs. The model achieved 93.21% accuracy for binary classification and 92.9% for multiclass, outperforming standalone CNNs while justifying the higher computational cost with improved detection of complex attacks. Acharya et al.^31^ proposed a CNN-BiLSTM hybrid IDS, with CNN for feature extraction and BiLSTM capturing both forward and backward temporal context. Evaluated on NSL-KDD and UNSW-NB15, it achieved 98.27% and 99.87% accuracy, respectively, with F1-scores over 98%, outperforming pure CNN or LSTM and excelling at detecting probe and U2R attacks.

Maddu and Rao^32^ proposed a deep learning IDS for SDN using NSL-KDD, employing a multi-layer architecture with optimized hyperparameters. The model achieved 98.88% accuracy and an average F1-score above 97%, showing strong detection performance for DoS and Probe attacks. A proposal by Aldallal^33^ combined a denoising autoencoder for unsupervised feature learning with a DNN for classification on KDDCup99. The hybrid IDS achieved 95 detection accuracy, showing that unsupervised representation learning improves the detection of subtle anomalies missed by purely supervised models.

Wang et al.^34^ proposed an industrial IDS framework for ICS, validated on NSL-KDD using KDDTest+ and KDDTest-21 subsets. The system achieved 84.25% accuracy on KDDTest+ but dropped to 69.42% on KDDTest-21, which includes novel attacks unseen during training. Another approach by Bin et al.^35^ is ADFCNN-BiLSTM, which combines attention-driven CNN feature extraction with BiLSTM temporal modeling. Evaluated on NSL-KDD and UNSW-NB15, it achieved a 97.23% detection rate and a very low 0.01% false positive rate. Hnamte and Hussain^36^ compared BiLSTM-CNN and CNN-BiLSTM on NSL-KDD, finding CNN-BiLSTM slightly better for binary classification, while BiLSTM-CNN achieved higher multiclass precision (82.91%) and excelled at rare classes like U2R and R2L. Both hybrids maintained overall accuracies above 90%, showing that model ordering affects the capture of sequential versus spatial features.

Alsubaei^37^ enhanced LSTM architectures with semantic attention to emphasize key feature subsets. The system achieved 82.21% accuracy and an F1-score of 82.27% on NSL-KDD, showing balanced performance across categories. Singh et al.^38^ proposed a wide-and-deep transfer learning framework with a stacked GRU network, using wide components for memorizing frequent patterns and deep components for generalization. The model achieved 99.92% accuracy on KDDCup99-10% and 94.22% on UNSW-NB15 multiclass classification.

Acharya et al.^39^ proposed a CNN-BLSTM hybrid, with CNN layers for hierarchical feature extraction and BiLSTM for temporal context learning. The system achieved 95.4% accuracy on NSL-KDD, improved detection of complex attacks like R2L, and balanced precision and recall, highlighting its promise for deployment-ready IDS. Cao et al.^40^ proposed a CNN–GRU hybrid, using CNN for spatial feature extraction and GRU for modeling temporal dependencies in network traffic. Evaluated on NSL-KDD and UNSW-NB15, it achieved 99.69% and 86.25% accuracy, respectively, outperforming traditional ML methods and demonstrating robustness against class imbalance and feature redundancy.

Sayegh et al.^41^ optimized LSTM-based IDS architectures by evaluating different optimizers and hyperparameters on UNSW-NB15. Using advanced optimizers like Nadam and AdaBelief, they achieved  97% accuracy with greater stability and reduced training time, highlighting how optimizer choice can significantly impact LSTM IDS performance. Benaddi et al.^42^ proposed a lightweight hybrid IDS for IoT, combining shallow CNN layers with lightweight RNN cells to balance accuracy and efficiency. Evaluated on NSL-KDD and UNSW-NB15, it achieved mid-90% accuracy with lower memory and processing demands, making it suitable for resource-constrained edge environments despite slightly lower accuracy than heavyweight deep models.

Dash et al.^43^ optimized LSTM-based IDS training using the Nadam optimizer on KDDCup99 and NSL-KDD, achieving 97.54% accuracy, 98.95% detection rate, and 9.98% false alarm rate. The results highlight that LSTM performance depends heavily on learning dynamics, with Nadam enabling faster convergence and reduced oscillations compared to Adam or RMSProp. Ibrahim and Elhafiz^44^ proposed an LSTM-RNN IDS with careful preprocessing, including encoding, scaling, and class balancing, achieving mid-90s overall accuracy on NSL-KDD and reducing false positives compared to simpler RNNs. The model improved per-class detection for Probe and DoS attacks, highlighting LSTM’s strength in modeling temporal correlations, with ablations on embedding size and depth addressing overfitting concerns. Devendiran and Turukmane^45^ introduced Dugat-LSTM, enhancing LSTMs with chaotic feature transforms to enrich inputs before temporal modeling, achieving 95% overall accuracy on NSL-KDD. The approach improved detection of minority classes and reduced false negatives, though it introduced computational overhead, with recommendations provided for real-time deployment. Umer et al.^46^ proposed a hybrid Transformer encoder + BiLSTM IDS with wrapper-based feature selection, achieving mid-90s multiclass accuracy on UNSW-NB15 and NSL-KDD. The Transformer captures long-range dependencies while BiLSTM models local temporal context, combining global attention with recurrent smoothing for robust IDS performance.

Hnamte and Hussain^47^ proposed a lightweight CNN-BiLSTM IDS for resource-constrained deployment, achieving low-to-mid 90s binary and multiclass accuracy on UNSW-NB15. Farhan et al.^48^ evaluated a deep neural network IDS across both UNSW-NB15 and NSL-KDD, reporting detection accuracies of 89.85% and 88.95%, respectively. These numbers reflect stronger performance than many traditional ML approaches, although they lag behind some optimized RNN hybrids. Liu et al.^49^ also analyzed the robustness of RNN-based IDS against adversarial sequences using NSL-KDD. They reported that their adversarial attacks increased the misclassification rate to over 96.68% across attack categories under both white-box and black-box settings–revealing striking vulnerability in RNN models. Shone et al.^50^ proposed a deep learning approach for intrusion detection, where a nonsymmetric deep autoencoder (NDAE) for unsupervised feature learning was provided. In addition, they introduced a deep learning classification model built using stacked NDAEs. Their classifier was implemented in GPU-enabled TensorFlow and evaluated using the benchmark KDD Cup’99 and NSL-KDD datasets.

Yang et al.^51^ adopted a systematic literature review methodology to survey and analyze 119 highly cited papers on anomaly-based intrusion detection. Their study provides a rigorous and comprehensive examination of the field’s technical landscape, intending to support and guide future research. Tian et al.^52^ also investigated multi-label adversarial example attacks on multi-label “false data injection attacks (FDIA)” locational detectors and introduced a general multi-label adversarial attack framework, called “muLti-labEl adverSarial falSe data injectiON (LESSON)”. This framework integrates three core design strategies: a) state variable perturbation, b) customized loss function design, and c) variable transformation, to identify effective multi-label adversarial perturbations that comply with physical constraints while bypassing both “Bad Data Detection (BDD)” and “Neural Attack Location (NAL)”. Moreover, Tian et al.^53^ proposed joint “adversarial examples and false data injection attacks (AFDIAs)” to investigate different attack scenarios against state estimation in power systems. Since perturbations applied directly to measurements are more likely to be detected by BDDs, their approach introduces perturbations to state variables, thereby ensuring that the attack remains stealthy with respect to BDDs. Additionally, Tian et al.^54^ proposed both a signal-specific approach and a universal signal-agnostic approach for attacking power systems using generated adversarial examples. They also introduced and evaluated black-box attacks that exploit transferability characteristics. In addition, adversarial training was employed as a defense mechanism to enhance system robustness against adversarial attacks.

#### Research gap

While the aforementioned studies demonstrate promising results in developing IDS systems, certain limitations persist that restrict their real-world effectiveness:

1. *Low accuracy on minority classes:* Most models perform well on high-frequency attack types such as DoS, but struggle to achieve high accuracy on low-frequency classes like U2R and R2L. This often stems from using shallow models incapable of extracting sufficient discriminative features.
2. *Feature reduction impact:* Although feature reduction or dimensionality reduction improves efficiency, it may inadvertently remove important predictive attributes. This can degrade performance in multi-class scenarios where subtle feature interactions matter.
3. *Simplified classification:* Some studies reduce the classification task to a binary problem – attack vs. normal, ignoring the need to distinguish between multiple types of attacks. This limits their applicability in realistic and complex threat landscapes.

Most existing intrusion detection studies primarily focus on single-dataset evaluations, often combined with aggressive feature reduction techniques or highly complex deep learning architectures aimed at maximizing overall accuracy. While these approaches can yield strong results on specific benchmarks, they frequently limit cross-dataset generalizability, reduce interpretability, and make fair comparison across studies difficult. Moreover, several works emphasize aggregate performance metrics, which provide limited insight into minority and low-frequency attack classes. These are critical in real-world intrusion detection scenarios. As a result, there remains a gap in the literature for systematic, reproducible evaluations that assess the behavior of standard deep learning models across multiple benchmark datasets using consistent experimental settings and complete feature representations.

To address this gap, this paper presents a comprehensive empirical analysis of DNN and RNN models evaluated on three widely used intrusion detection datasets (KDDCup99, NSL-KDD, and UNSW-NB15) under identical preprocessing, training, and evaluation protocols. Unlike feature-reduction–based approaches, we analyze the impact of full-feature learning on detection performance, with particular emphasis on per-class precision, recall, and F1-score to better understand minority attack detection. Furthermore, this study emphasizes reproducibility by explicitly reporting model configurations, hyperparameters, and training settings, enabling fair comparison and facilitating future research. The deep learning-based models leverage the full feature set and are trained using GPU-accelerated environments to ensure scalability and accuracy. By employing deep feedforward and recurrent architectures, we aim to enhance detection performance across all attack categories, including those with fewer examples. In summary, while existing approaches to intrusion detection show notable progress, key challenges such as improving multi-class accuracy and minimizing false positives remain.

## Proposed methodology

The considered models are DNN and RNN, which are trained on input features and output labels to learn predictive patterns. To function effectively, they require a loss function to measure the gap between predictions and true outputs, along with an optimizer to adjust weights and biases. The choice of optimizer and loss function varies by model. Implemented in PyTorch, these models operate on tensors, multidimensional numerical arrays, whereas the datasets are in CSV format, containing non-numeric features. Therefore, before training, the data must be transformed into a compatible numerical structure. This essential process, known as data preprocessing, ensures the model can interpret and learn from the inputs without conflict.

### Pre-processing of data

The data we have in our CSV files will be read into a Pandas DataFrame without any errors. But if we try to run this data through our model, it will spark a few issues. The main issue is that our Deep Learning Model is not developed to take the string datatype values. So to avoid this, Data has to be encoded.

For our purpose, Label Encoder has provided great results. Label Encoder converts categorical string labels into numerical values. The classes in our dataset go from string to integers, like $0, 1, 2, \ldots , N-1$ (*N* here being the number of output labels). Even after doing the encoding, we feel like the data is not properly scaled. For this, we need to perform normalization or Scaling. For this work, we found that the StandardScaler from scikit-learn provided the best results possible.

**Fig. 1:**
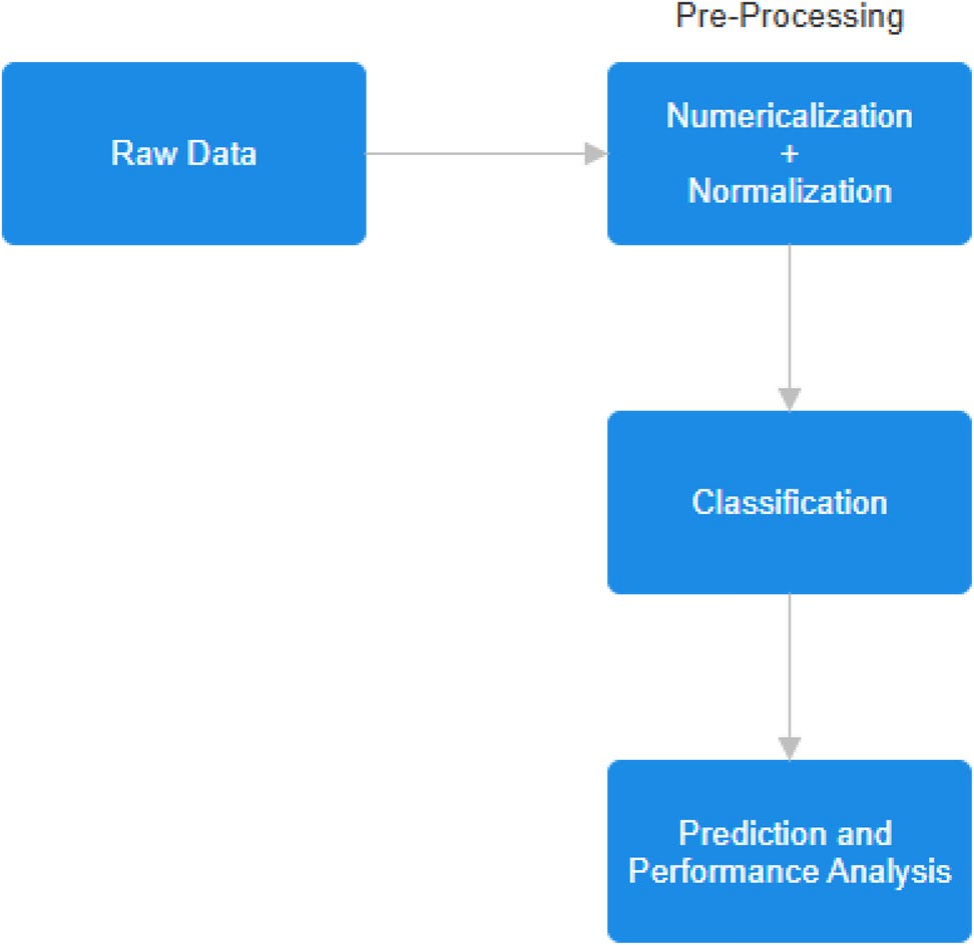
Flowchart of proposed methodology.

The Label Encoder and StandardScaler are used in the procedure. The LabelEncoder’s job is not to actually describe any order (or priority). Its job is to assign numbers to the values (basically convert the distinct values to numeric data, so the models can process them better). One-hot encoding is another option, but the number of features it adds onto the existing feature list, which leads to extra in-features, is something that made us choose LabelEncoder. The missing values are not a matter of concern, as the three datasets employed(NSL-KDD, KDDCup99, and UNSW-NB15) have no missing values that need to be addressed in any columns.

Specifically, ReLU activation was used for all models due to its computational efficiency and stable gradient propagation during training. Compared to saturating activation functions, such as sigmoid or tanh, ReLU mitigates vanishing gradient effects and typically enables faster and more reliable convergence. Using a consistent activation function across both DNN and RNN models also ensured a fair and controlled comparison between architectures. The number of layers and neurons was selected through preliminary experimentation to achieve a balance between model capacity and training efficiency. Deeper or wider architectures did not yield consistent performance gains and led to increased training time and overfitting tendencies. Therefore, the final configurations were chosen to maximize accuracy while maintaining computational efficiency.

Figure 1 shows the procedure of the proposed methodology. The goal of this paper is to perform the following tasks:

1) To enhance DNN and RNN models and implement them using a Deep Learning (DL) framework.

2) To train and test these models on datasets, like KDDcup99, NSL-KDD, and UNSW-NB15.

3) To find out the accuracy of every attack on the datasets.

### Architecture of DNN and RNN

#### Enhanced DNN architecture

To effectively classify network connections into multiple attack categories, we propose a deep feedforward neural network built using the nn.Sequential module in PyTorch. This architecture was selected for its simplicity, computational efficiency, and ability to generalize well across structured tabular data commonly found in network intrusion detection datasets.

The architecture is designed to support multiple intrusion detection datasets with different feature dimensions. The input layer adapts based on the dataset, specifically:

1. KDDCup99: 40 input features
2. NSL-KDD: 40 input features
3. UNSW-NB15: 42 input features

The network architecture remains consistent across datasets, comprising three fully connected layers with ReLU activations. The structure is as follows:

1. *Input layer*: Maps raw features (40 or 42) to a 128-dimensional hidden representation.
2. *Hidden layer 1*: Dense layer with 128 units and ReLU activation, introducing non-linearity for pattern learning.
3. *Hidden layer 2*: Another dense layer with 128 neurons and ReLU activation, enhancing depth for complex feature extraction.
4. *Output layer*: Fully connected layer reducing to 5 neurons (Normal, DoS, Probe, R2L, U2R) with softmax for class probabilities.

The model can be summarized as:

$$\hat{y} = \text {Softmax} \left( W_3 \cdot \text {ReLU} \left( W_2 \cdot \text {ReLU} \left( W_1 \cdot x + b_1 \right) + b_2 \right) + b_3 \right)$$

where $x \in \mathbb {R}^{d}$ is the input vector with $d \in \{40, 42\}$, $W_i$ and $b_i$ are the weights and biases of the $i^\text {th}$ layer, and $\hat{y}$ is the predicted probability distribution over the five classes.

This architecture mitigates overfitting by using a shallow yet sufficiently deep structure to capture non-linear patterns. Training employs cross-entropy loss with the Adam optimizer, leveraging adaptive learning rates and momentum. Key hyperparameters–batch size, learning rate, and epochs–are tuned via validation. Overall, the model acts as a general-purpose, dataset-agnostic classifier for benchmarking intrusion detection.

#### Enhanced RNN-based model architecture

To capture temporal dependencies and sequential patterns in network traffic, a Recurrent Neural Network (RNN)-based model is proposed. Unlike feedforward networks that treat features independently, RNNs can exploit the order and interaction between features such as duration, bytes, flags, and protocol type–crucial for distinguishing attack signatures in intrusion detection.

The model is implemented in PyTorch as a flexible class, dynamically adjusting input features and output classes based on the dataset:

1. *KDDCup99/NSL-KDD:* 40 features, 5 output classes (*Normal, DoS, Probe, R2L, U2R*).
2. *UNSW-NB15:* 42 features, 10 output classes (*Fuzzers, Reconnaissance, Backdoors, Shellcode*, etc., plus normal traffic).

The architecture consists of the following:

1. *Input layer:* Vector of size 40/42 reshaped with a temporal dimension using x.unsqueeze(1).
2. *Recurrent layer:* Single-layer vanilla RNN with hidden size 64, ReLU activation, and batch_first=True. Since sequence length is 1, only the final hidden state is used.
3. *Fully connected layer:* Maps the 64-dimensional hidden state to the number of output classes.
4. *Output activation:* Softmax applied to logits during inference to generate class probabilities.

The forward propagation logic of the model could be described as follows:

1. The input vector $x \in \mathbb {R}^{B \times F}$, where $B$ is our batch size and $F \in \{40, 42\}$ is our feature size, is reshaped to $\mathbb {R}^{B \times 1 \times F}$ to simulate a temporal sequence of length 1.
2. The reshaped input is passed through the RNN layer, yielding a sequence of hidden states. Since the sequence length is 1, the final hidden state $h_T \in \mathbb {R}^{B \times H}$ (where $H = 64$) is selected for classification.
3. The hidden state is sent to a fully connected layer to produce the logits $\hat{y} \in \mathbb {R}^{B \times C}$, where $C \in \{5, 10\}$ is the number of output classes.

Mathematically, this can be shown as:

$$\hat{y} = W_{fc} \cdot h_T + b$$

where $W_{fc}$ and $b$ are the parameters of the final linear layer, and $h_T$ is the last hidden state output by the RNN. This model is particularly lightweight and efficient, making it suitable for rapid experimentation on intrusion detection tasks. Although only a single-layer RNN is used here, the design can be extended to multi-layer RNNs or more complex recurrent architectures (e.g., LSTM or GRU) for deeper temporal modeling. Overall, the model offers a strong balance between simplicity and effectiveness, especially for datasets where features represent implicit time-structured behavior.

Here, the datasets used in this study are tabular, record-based datasets rather than temporal sequence data. As such, there are no explicit long-term temporal dependencies that need to be modeled. Consequently, the vanishing gradient issue commonly associated with vanilla RNNs in long-sequence modeling is not a limiting factor in this setting. In this work, the RNN is employed to capture feature-level interactions rather than temporal dependencies across network flows. Therefore, the choice of a vanilla RNN was considered sufficient and appropriate for the characteristics of the data. The observed performance on UNSW-NB15 is more closely related to class imbalance and inter-class similarity within the dataset, rather than limitations arising from long-term dependency modeling.

The “full features” have been found to yield better results in comparison to dimensionality reduction. This suggests that all the features are necessary for developing a better model and there are no throw-away features present in these datasets. It is worth noting that the three datasets used in this study (KDDCup99, NSL-KDD, and UNSW-NB15) are tabular, record-based datasets rather than sequential time-series data. As a result, long temporal dependencies are not explicitly present, and therefore, vanishing gradient issues typically associated with long sequence modeling are unlikely to arise in this setting. This motivates us to choose a simple vanilla RNN, which is effective for feature-based intrusion detection tasks^55^.

Regarding architectural complexity, we initially experimented with higher-complexity models, including a tabular transformer architecture. However, these models yielded lower detection accuracy and reduced generalization, which indicates that such complex architectures may be over-parameterized for record-based IDS data. Similar observations have been reported in prior studies, where simpler deep learning models demonstrated competitive or superior performance on tabular intrusion datasets^50^. Based on these findings, we deliberately adopted a simplified DNN and a vanilla RNN to better match the characteristics of the data. Empirical results show that these models achieved more stable training and improved performance compared to more complex architectures.

For NSL-KDD, we use the KDDTrain+ dataset, which is divided into mutually exclusive training and testing subsets using an 80:20 split. Similar splitting procedures are applied consistently across KDDCup99 and UNSW-NB15 to ensure uniform experimental conditions. As the datasets employed are preprocessed and synthetically generated, the resulting training and testing subsets follow the same underlying distribution while remaining statistically independent. This approach allows for controlled evaluation of model behavior under consistent conditions and supports fair cross-dataset comparison. Moreover, regarding baseline comparisons, our study focuses on internal consistency and reproducibility by evaluating all models under the same preprocessing, feature sets, and training protocols. Instead of re-implementing external baselines, we compare our findings with standardized performance ranges reported in recent surveys and benchmark studies.

### Output encoding

In supervised learning tasks such as intrusion detection, categorical output labels must be converted into numerical form for processing by ML and DL models. Neural networks, in particular, require both input features and target labels to be numeric. To achieve this, we apply label encoding, which maps each class to a unique integer. This is implemented using the LabelEncoder class from the scikit-learn library. The assigned integers are consistent and deterministic, with the exact mapping varying by dataset and depending on whether the task is binary or multi-class classification.

For binary classification tasks, which involve distinguishing between normal and attack traffic, the labels are encoded as: Normal $\rightarrow$ 0, Attack $\rightarrow$ 1. This format is particularly used when evaluating the overall ability of the model to distinguish benign from malicious activity, without regard to specific attack types. In multi-class classification, where the objective lies in identifying a specific type or category of an attack, a different encoding scheme is applied.

The integer encoding corresponds to the number of distinct attack categories in the dataset:

KDDCup99 and NSL-KDD:

$$\texttt {Normal} \rightarrow 0, \quad \texttt {DoS} \rightarrow 1, \quad \texttt {Probe} \rightarrow 2, \quad \texttt {R2L} \rightarrow 3, \quad \texttt {U2R} \rightarrow 4$$

UNSW-NB15:

$$\begin{aligned} \begin{array}{ll} \texttt {Normal} \rightarrow 0, & \texttt {Fuzzers} \rightarrow 1, \\ \texttt {Analysis} \rightarrow 2, & \texttt {Backdoor} \rightarrow 3, \\ \texttt {DoS} \rightarrow 4, & \texttt {Exploits} \rightarrow 5, \\ \texttt {Generic} \rightarrow 6, & \texttt {Reconnaissance} \rightarrow 7, \\ \texttt {Shellcode} \rightarrow 8, & \texttt {Worms} \rightarrow 9 \end{array} \end{aligned}$$

The exact mapping may vary depending on the preprocessing order, but it remains consistent across training and evaluation once fitted. This numerical representation is crucial since classification models output logits or class probabilities. During training, the encoded labels are used with CrossEntropyLoss, which expects integer class indices. In inference, the model predicts an index that can be mapped back to the original label via the inverse transform of the encoder. Accurate label encoding ensures correct class interpretation in both training and evaluation, while also enabling compatibility with categorical metrics such as accuracy, precision, recall, and confusion matrices.

## Results and discussions

This section evaluates the enhanced DNN and RNN models on three benchmark datasets: KDDCup99, NSL-KDD, and UNSW-NB15. Performance is assessed using metrics such as accuracy, precision, F1-score, and False Alarm Rate (FAR), with results illustrated through six comparative graphs highlighting model performance across datasets.

### Performance on KDDCup99

**Fig. 2:**
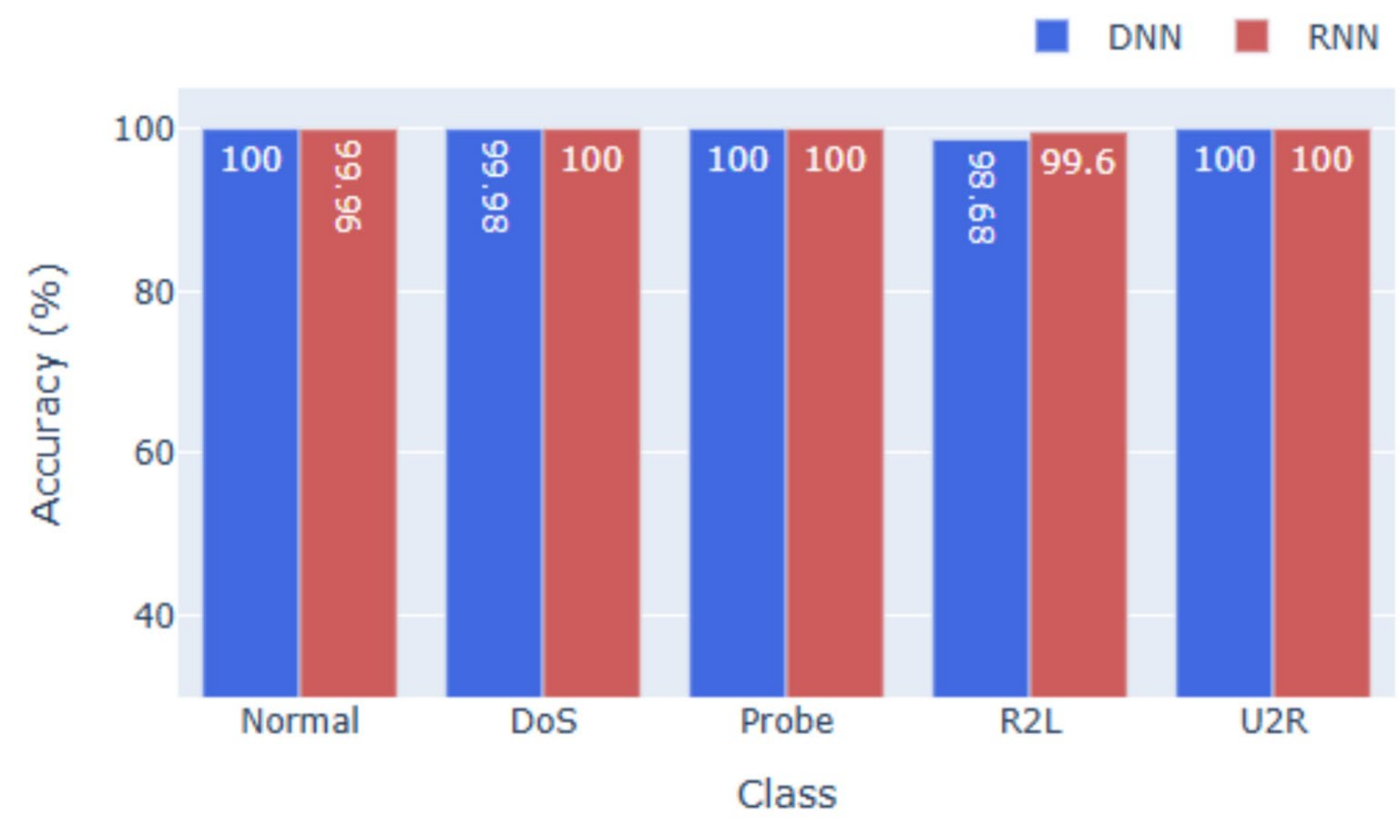
Accuracy for KDDcup99.

As shown in Figs. 2 and 5, both models achieve near-perfect results on KDDCup99. The DNN attains **99.98%** accuracy, while the RNN records **99.47%**, with equally high F1-scores reflecting balanced precision and recall. False alarm rates remain minimal, underscoring model robustness. The small performance gap indicates that the dataset’s tabular structure is effectively captured by DNNs, reducing the added value of RNNs for sequential modeling in this context.

### Performance on NSL-KDD

**Fig. 3:**
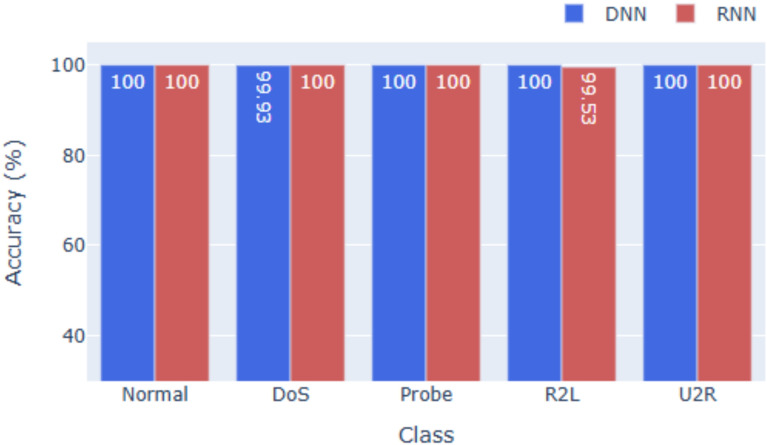
Accuracy for NSLKDD.

For NSL-KDD (see Figs. 3 and 6), the DNN achieves **99.96%** accuracy, while the RNN records **99.13%**. Precision and F1-scores remain high across all five categories, confirming balanced performance. Given NSL-KDD’s reduced redundancy and greater difficulty, these results highlight strong generalization. The slightly lower RNN accuracy suggests limited temporal dependencies, though both models still surpass traditional ML baselines reported in prior work.

### Performance on UNSW-NB15

**Fig. 4:**
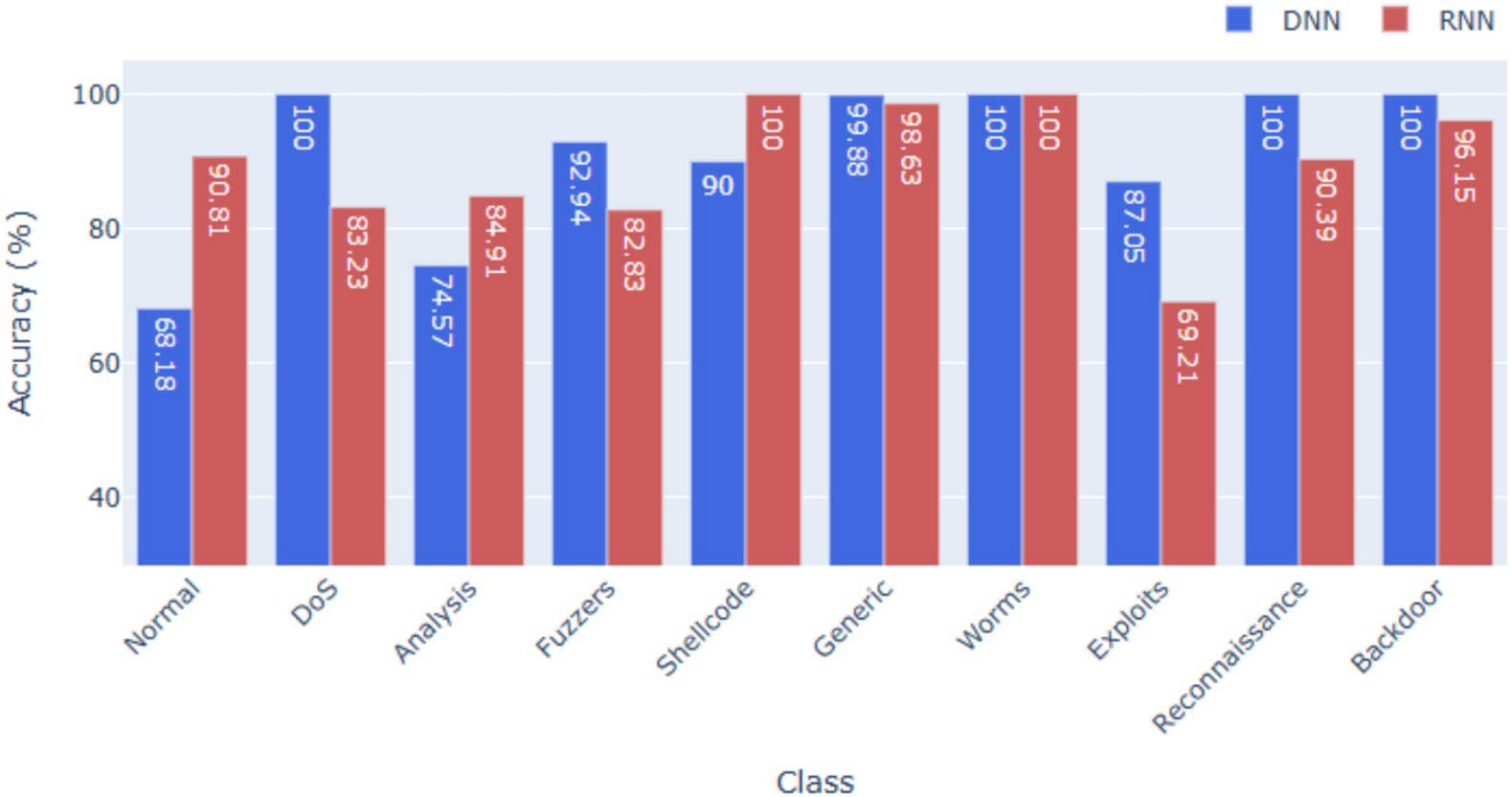
Accuracy for UNSW-NB15.

UNSW-NB15, a modern dataset with 10 diverse classes, presents greater complexity. As shown in Figs. 4 and 7, the DNN achieves **95.96%** accuracy, clearly outperforming the RNN at **82.00%**. This gap reflects the dataset’s subtle feature distinctions, which are better captured by DNNs with layered non-linear transformations, whereas a simple RNN struggles without deeper architectures. Across other metrics, the DNN also achieves higher precision and F1-scores with lower FAR, while the RNN’s weaker results point to the need for deeper or advanced recurrent variants (e.g., LSTM, GRU) to better handle heterogeneous traffic patterns.

### Cross-dataset insights

Across all three datasets, the DNN consistently outperforms the RNN in terms of overall accuracy, F1-score, and precision. This suggests that while RNNs are useful for sequential data, tabular intrusion detection datasets, especially those with limited temporal ordering, are better suited for deep feedforward networks. The models also demonstrate strong class-wise generalization. High F1-scores for low-frequency classes (e.g., U2R, R2L, Worms) indicate that the models are not overly biased toward majority classes, which is a common challenge in multi-class intrusion detection.

**Fig. 5:**
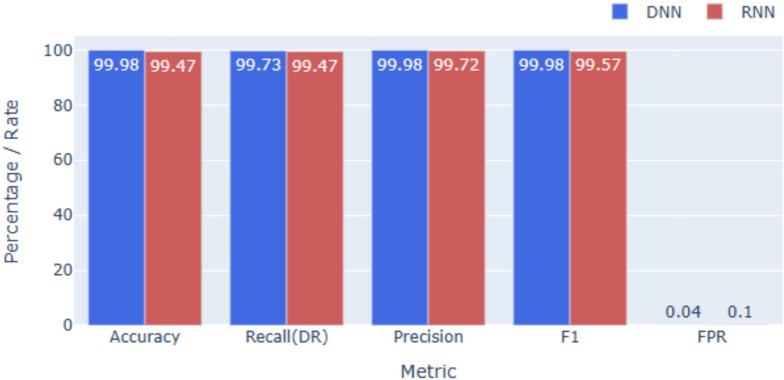
Overall performance on KDDcup99.

**Fig. 6:**
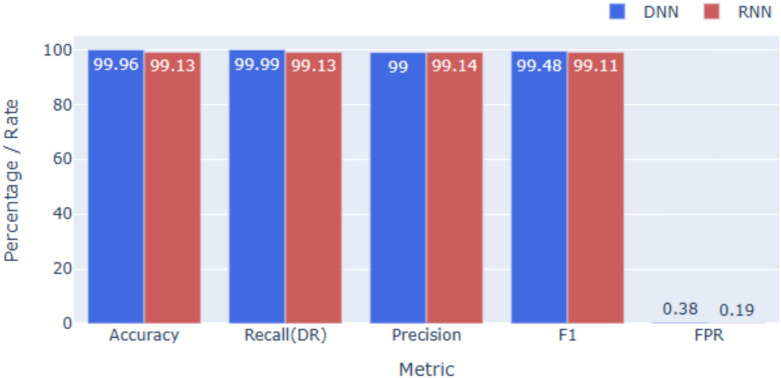
Overall performance on NSL-KDD.

**Fig. 7:**
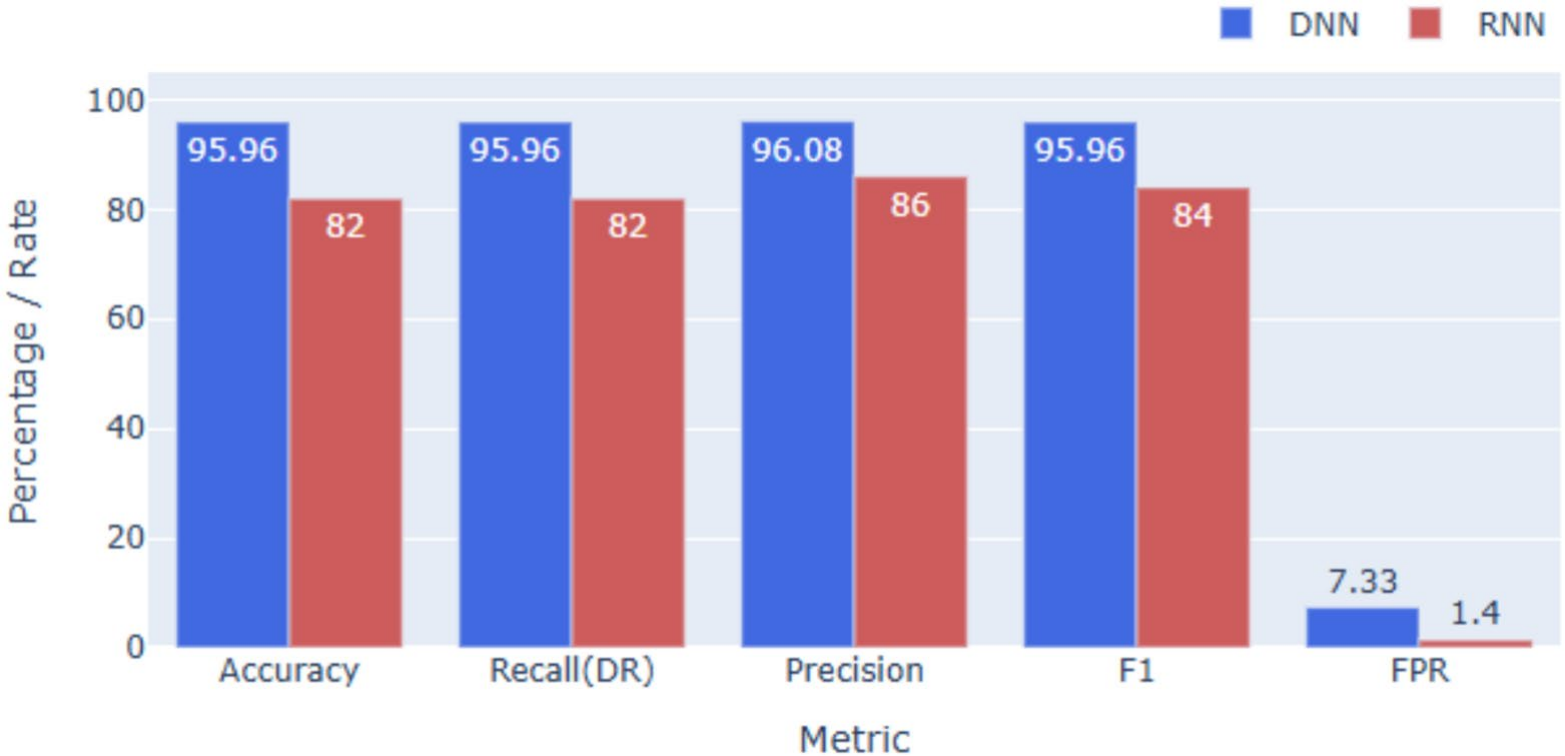
Overall performance on UNSW-NB15.

**Fig. 8:**
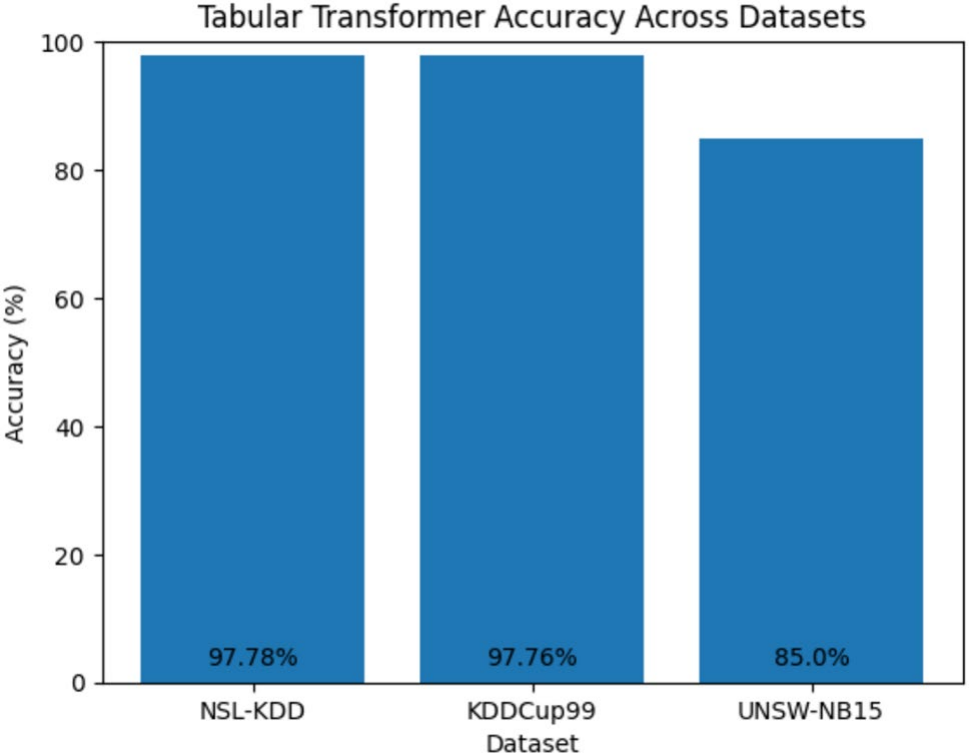
Accuracies of the transformer in different datasets.

It is well-known that advanced architectures, such as LSTM, GRU, hybrid CNN–RNN, and Transformer models, are effective for modeling complex sequential and temporal dependencies. However, the datasets used in this study (KDDCup99, NSL-KDD, and UNSW-NB15) are tabular, record-based datasets, rather than true sequential time-series data. As a result, long-term temporal dependency modeling is not a primary requirement in this setting, and the limitations of vanilla RNNs related to vanishing gradients are less pronounced.

To assess whether higher model complexity would yield performance gains, we conducted additional experiments using a tabular transformer model under the same experimental setup. The transformer achieved accuracies of 85% on UNSW-NB15, 97.78% on NSL-KDD, and 97.76% on KDDCup99, which did not consistently outperform the simpler DNN and vanilla RNN architectures used in this study (as shown in Fig. 8). These results indicate that increased architectural complexity does not necessarily translate to improved performance for record-based IDS datasets and may lead to over-parameterization. Based on these observations, the use of a simplified DNN and a vanilla RNN was a deliberate design choice.

## Comparative study

To assess the effectiveness of the proposed DNN and RNN architectures, we compared their performance against benchmark models previously applied to KDDCup99 and NSL-KDD. The evaluation focuses on classification accuracy to highlight improvements over traditional ML methods and earlier deep learning approaches.

### Comparison on NSL-KDD dataset

Table 1 presents a summary of different classification techniques applied to the NSL-KDD along with the reported accuracies. Several models, such as Naive Bayes, K-Means, and hybrid K-Means with Random Forest, have been investigated in prior studies. As shown in Table 1, both models outperform the previous best accuracy of 92.77% (K-Means + RF). The DNN attains 99.96%, while the RNN achieves 99.13%, confirming the strength of deep architectures in capturing complex patterns in network traffic.

**Table 1:** Comparison of classification methods and their accuracy on NSL-KDD.

**Method**	**Accuracy (%)**
Naive Bayes Classifier^8^	97.00
K-Means + RF^10^	92.77
K-Means^15^	82.29
RNN^20^	89.6
Deep Stacking Network^23^	86.8
LSTM^25^	87.8
K-means clustering^26^	82.19
CNN^27^	93.65
CNN-BiLSTM hybrid^31^	98.27
LSTM^43^	97.54
Dugat-LSTM^45^	95
**Enhanced DNN**	**99.96**
**Enhanced RNN**	**99.13**

### Comparison on KDDCup99 dataset

Table 2 lists several deep learning-based models evaluated on the KDDCup99 dataset. These include Autoencoders (AE) and the addition of Deep Belief Networks (DBN), and other hybrid systems. As shown in Table 2, the DNN achieves 99.98% accuracy, surpassing the previous best of 99.30% (DBN). The RNN attains 99.47%, also outperforming most existing methods, underscoring the effectiveness of the proposed deep models on legacy datasets.

**Table 2:** Comparison of classification methods and their accuracy on KDDCup99.

**Method**	**Accuracy (%)**
Deep Belief Network (DBN)^16^	99.30
DESC-IDS^17^	96.44
Autoencoder (AE)^18^	94.71
Denoising Autoencoder^33^	95
LSTM ^43^	97.54
**Enhanced DNN**	**99.98**
**Enhanced RNN**	**99.47**

### Discussion

The comparative analysis demonstrates that the proposed DNN and RNN consistently outperform both traditional and recent deep learning models across datasets, benefiting from their ability to learn complex feature relationships without manual engineering.

1. On NSL-KDD, the DNN improves accuracy by over 7% compared to the best traditional method (K-Means).
2. On KDDCup99, the DNN surpasses the DBN benchmark by 0.68%.
3. The RNN, while slightly less accurate than the DNN, still exceeds prior models.

These findings establish the proposed architectures as competitive solutions for real-world Network Intrusion Detection Systems (NIDS), providing both precision and scalability across diverse attack scenarios.

## Conclusion and future work

This study provides a comprehensive evaluation of two deep learning models, such as Deep Neural Networks (DNN) and Recurrent Neural Networks (RNN), for network intrusion detection using three benchmark datasets: KDDCup99, NSL-KDD, and UNSW-NB15. The results demonstrate that both models achieve high accuracy and low false positive rates, effectively classifying multiple categories of network attacks. Their strong performance can be attributed to the use of non-linear activation functions, carefully selected loss functions and optimizers, and the decision to retain the complete set of input features. Preserving the full feature space allowed the models to learn complex decision boundaries, thereby improving classification accuracy. High F1-scores across all datasets, including those with significant class imbalances, further validate the robustness and generalization capabilities of the models in identifying both frequent and rare attack types. Overall, these findings underscore the effectiveness of deep learning architectures that fully exploit available features, offering reliable, scalable, and high-performing solutions for real-world intrusion detection systems. The models effectively address the challenges of data complexity and class imbalance in next-generation network IDS deployments.

Although the deep learning-based IDS achieves strong performance, several enhancements could further improve its robustness and applicability in real-world environments. One promising direction is to extend the RNN architecture to more advanced recurrent models such as LSTM networks and “Gated Recurrent Units (GRUs)”, which can better capture long-term dependencies, particularly valuable for complex and imbalanced datasets like UNSW-NB15. Additionally, feature reduction techniques could be explored to improve computational efficiency without compromising accuracy. Methods such as “Principal Component Analysis (PCA)”, “Recursive Feature Elimination (RFE)”, and mutual information-based filtering can remove redundant or less informative features, thereby reducing training time, lowering memory usage, and improving interpretability. Moreover, hybrid classification approaches could yield further performance gains. Employing DNNs for feature extraction followed by traditional classifiers such as “Random Forests”, “Support Vector Machines (SVMs)”, or boosting algorithms may balance deep representational learning with precise classification, adapting more effectively to diverse network traffic patterns. Collectively, these future directions have the potential to create more adaptive, efficient, and explainable intrusion detection systems, well-suited for modern cybersecurity environments where both accuracy and resource optimization are essential.

## Data Availability

The datasets generated and/or analysed during the current study are available in the kaggle.com/datasets repository: https://www.kaggle.com/datasets/galaxyh/kdd-cup-1999-data; https://www.kaggle.com/datasets/hassan06/nslkdd; https://www.kaggle.com/datasets/mrwellsdavid/unsw-nb15
